# A high resolution radiation hybrid map of bovine chromosome 14 identifies scaffold rearrangement in the latest bovine assembly

**DOI:** 10.1186/1471-2164-8-254

**Published:** 2007-07-26

**Authors:** Elisa Marques, Simon de Givry, Paul Stothard, Brenda Murdoch, Zhiquan Wang, James Womack, Stephen S Moore

**Affiliations:** 1 Department of Agricultural, Food and Nutritional Science, University of Alberta, Edmonton, AB, T6G 2P5, Canada; 2 Unité de Biométrie et d'Intelligence Artificielle INRA, BP 52627, 31326 Castanet-Tolosan, France; 3 Department of Veterinary Pathobiology, Texas A & M University, College Station, TX 77843, USA

## Abstract

**Background:**

Radiation hybrid (RH) maps are considered to be a tool of choice for fine mapping closely linked loci, considering that the resolution of linkage maps is determined by the number of informative meiosis and recombination events which may require very large mapping populations. Accurately defining the marker order on chromosomes is crucial for correct identification of quantitative trait loci (QTL), haplotype map construction and refinement of candidate gene searches.

**Results:**

A 12 k Radiation hybrid map of bovine chromosome 14 was constructed using 843 single nucleotide polymorphism markers. The resulting map was aligned with the latest version of the bovine assembly (Btau_3.1) as well as other previously published RH maps. The resulting map identified distinct regions on Bovine chromosome 14 where discrepancies between this RH map and the bovine assembly occur. A major region of discrepancy was found near the centromere involving the arrangement and order of the scaffolds from the assembly. The map further confirms previously published conserved synteny blocks with human chromosome 8. As well, it identifies an extra breakpoint and conserved synteny block previously undetected due to lower marker density. This conserved synteny block is in a region where markers between the RH map presented here and the latest sequence assembly are in very good agreement.

**Conclusion:**

The increase of publicly available markers shifts the rate limiting step from marker discovery to the correct identification of their order for further use by the research community. This high resolution map of bovine chromosome 14 will facilitate identification of regions in the sequence assembly where additional information is required to resolve marker ordering.

## Background

Radiation hybrid (RH) mapping has proved to be a powerful tool for establishing marker order across a number of species [1-3]. The advantage of RH mapping over other mapping approaches such as linkage maps is that RH mapping does not require polymorphic markers, therefore increasing the number of loci potentially mapped.

In 2005, Everts-van der Wind *et al*. [4] published the most comprehensive bovine whole genome radiation hybrid map including a total of 3000 markers on 29 chromosomes. Since then, two other genome wide RH maps [5,6] with additional markers have been released confirming the usefulness of RH maps. Considering that linkage maps are only useful when there is recombination between markers, a higher resolution RH panel provides the means to order closely linked markers. In addition, RH maps can be used either as scaffolds for correct genome assembly or for identifying and resolving misassembled regions of the genome sequence [7]. Currently, there are four whole genome radiation hybrid panels available in cattle [1,2,8,9], with the highest resolution (12 K rad) developed by Rexroad *et al*. [8].

The increased availability of markers has led to the development of new methods for RH data analysis and map construction. The comparative mapping approach, a newly incorporated algorithm in CarthaGene [10], takes advantage of the information already available for a particular genome assembly, building more robust maps than the traditional approach. Using simulated data, less than 10% of the markers were wrongly positioned using the comparative mapping approach, while 33% of incorrectly positioned markers were observed using the traditional RH approach [11]. The traditional RH approach relies on heuristic methods resulting in framework maps that include only a small portion of all the markers (20% to 50%) [11]. On the other hand, the comparative mapping approach extends the usual statistical model describing the RH data [12] by adding a non-uniform prior distribution on the possible orders. Overall, the comparative mapping approach exploits the knowledge of a completely sequenced genome containing markers that have orthologous relationships with markers genotyped through the RH panel [10,11].

Our study uses this new mapping algorithm to build a high resolution radiation hybrid map of bovine chromosome 14 (BTA14) comparing specific discrepancies between our map and the latest sequence assembly. The identification of the correct order of markers on a specific chromosome is essential to the research community. Specifically, the large number of carcass fatness quantitative trait loci (QTL) on BTA14 [13,14] makes it a prime target for fine scale mapping.

## Results

### Radiation hybrid map

A total of 843 single nucleotide polymorphism (SNP) markers were mapped to bovine chromosome 14 using the 12 K rad bovine whole genome radiation panel [8]. The majority of the SNP markers are derived from the bovine sequence database [15]. Twenty-four had been previously mapped using the 3 K panel, 64 are from unmapped bac end sequences (BES) and 3 are from within genes known to be on BTA14. The RH map obtained has a log10-likelihood of -3835.03, with a total length of 4690.3 centirays (cR) and an average marker spacing of 96 Kbp. The average retention frequency for all the markers mapped to BTA14 was 18%, with 478 unique retention patterns (Additional file 2). A list of all mapped markers and their respective RH positions and genotypes are given in additional file 1 and additional file 3.

### Alignment with RH_3,000 _BTA14 map

**Figure 1:**
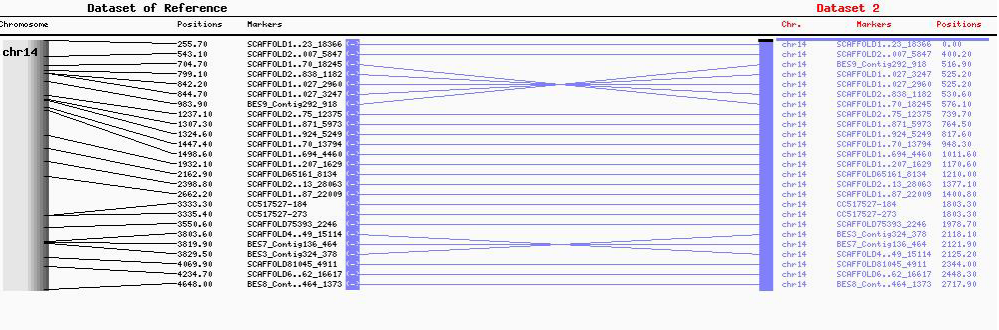
**12 K RH map of BTA14 compared with the UofA RH_3,000_**. The right side map refers to 12 K BTA14 while the left side refers to 3 K BTA14. Common markers are highlighted in bold and connected through blue lines. Distances on both maps are represented in cR.

There are 25 common markers between the high resolution BTA14 map presented here and the BTA14 *RH*_3,000 _map described in McKay *et al*. [6]. Overall, there is a high consistency in marker order, except for two regions where closely mapped markers are inverted (Figure 1). The first inconsistency is comprised of markers SCAFFOLD105570_18245, SCAFFOLD230838_1182, SCAFFOLD135027_2960, SCAFFOLD135027_3247 and BES9_contig292_918. In our map, their positions range from 543.1 to 844 cR. The other region involves three flanking markers showing an inversion in their positions: SCAFFOLD40049_15114 at 3803.6 cR, BES7_Contig136_464 at 3819.9 cR and BES3_Contig324_378 at 3829.5 cR

### Alignment with bovine sequence assembly (Btau_3.1)

**Figure 2:**
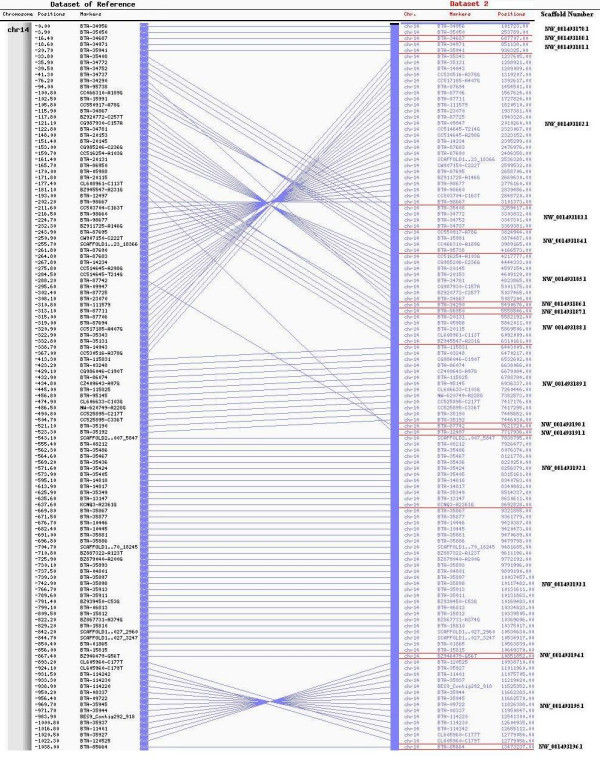
**12 K RH map of BTA14 compared to the corresponding Btau_3.1 map**. This figure shows the upper quartile map of the map. For the full image see additional file 5. The right side map refers to 12 K BTA14 while the left side map refers to Btau_3.1. Common markers are connected through blue lines. For legibility purposes, only markers with unique retention patterns are displayed. Distances on 12 K RH map are represented in cR, on the Btau_3.1 map in base pair positions. Red lines represent breaks in the scaffold numbers.

Of the 843 markers mapped, 20 had multiple hits on different chromosomes when compared to the bovine sequence assembly (Btau_3.1) using BLAST. Most of these hits occurred between BTA14 and an unassigned chromosome with similar BLAST scores (Table 1). There are several regions of discrepancies between our BTA14 RH map and the bovine sequence assembly (Btau_3.1) (Figure 2 and additional file 5). A major region of inconsistency is near the centromere with smaller regions throughout the chromosome showing flips between sets of markers. Overall, the inconsistencies can be summarized by:

**Table 1:** List of specific markers mapped to 12 K RH BTA14 map with BLAST hits to multiple chromosomes on Btau_3.1

**NCBI Accession number/NCBI SNP id**	**Marker name**	**BTA14 RH_12,000 _position (cR)**	**NCBI Accession number (scaffold)/BTA**	**BLAST Score/E-value**
ss61534608	BTA-34290	76.2	NW_001502201.1|BtUn_WGA3528_3 NW_001493186.1|Bt14_WGA1694_3	920 0.0
ss69374948	CC513828-C70T	313.1	NW_001497844.1|BtUn_WGA12210_3 NW_001493182.1|Bt14_WGA1690_3	279 3e-73
ss69374951	CC517185-A407G	320.9	NW_001508604.1|BtUn_WGA9931_3 NW_001493182.1|Bt14_WGA1690_3	361 2e-97
ss69374954	CC517185-A286G	338.7	NW_001508604.1|BtUn_WGA9931_3	366 5e-99
ss61535184	BTA-35317	413.3	NW_001504912.1|BtUn_WGA6239_3	920 0.0
ss69374970	BZ879040-A200G	725.9	NW_001508460.1|BtUn_WGA9787_3 NW_001493193.1|Bt14_WGA1701_3	366 5e-99
ss61473730	BTA-114222	938.9	NW_001493195.1|Bt14_WGA1703_3 NW_001495461.1|Bt8_WGA1109_3	894 0.0
ss38334682	BTA-12630	1705.8	NW_001493441.1|Bt16_WGA1949_3 NW_001493205.1|Bt14_WGA1713_3	920 0.0
ss61480494	BTA-34395	1847.2	NW_001503381.1|BtUn_WGA4708_3	754 0.0
ss61480492	BTA-34393	1847.2	NW_001503381.1|BtUn_WGA4708_3	769 0.0
ss61508240	BTA-34679	2593.8	NW_001493216.1|Bt14_WGA1724_3 NW_001505586.1|BtUn_WGA6913_3	920 0.0
ss61508239	BTA-34678	2593.8	NW_001493216.1|Bt14_WGA1724_3 NW_001505586.1|BtUn_WGA6913_3	920 0.0 915 0.0
ss61508238	BTA-34677	2593.8	NW_001505586.1|BtUn_WGA6913_3 NW_001493216.1|Bt14_WGA1724_3	920 0.0
ss61508235	BTA-34674	2606.1	NW_001505586.1|BtUn_WGA6913_3 NW_001493216.1|Bt14_WGA1724_3	887 0.0
ss61475994	BTA-16955	2767.3	NW_001497831.1|BtUn_WGA12197_3 NW_001493220.1|Bt14_WGA1728_3	915 0.0
ss61485958	BTA-55549	3011.7	NW_001494573.1|Bt2_WGA221_3 NW_001493224.1|Bt14_WGA1732_3	920 0.0 898 0.0
ss38337066	BTA-15014	3271.8	NW_001502681.1|BtUn_WGA4008_3	915 0.0
ss61564952	BTA-90430	3343.8	NW_001507724.1|BtUn_WGA9051_3 NW_001493239.1|Bt14_WGA1747_3	512 6e-143
ss61547746	BTA-58540	3401.2	NW_001494258.1|Bt24_WGA2566_3 NW_001493238.1|Bt14_WGA1746_3	869 0.0 867 0.0
ss61467380	BTA-35166	3679.4	NW_001493243.1|Bt14_WGA1751_3 NW_001493244.1|Bt14_WGA1752_3	920 0.0

A) Single markers or group of closely mapped markers mapping somewhere else in the bovine sequence assembly. ie: BTA-12497 and BTA-20131 to BTA-86950

B) Inversion of flanking markers. ie: BTA-11589 and BTA-34555

C) Inversion of closely mapped markers. ie: BTA-04776 to BTA-06606

All inversions between closely mapped markers were analyzed and suggest incorrectly ordered scaffolds. The first case is represented by markers BTA-20131 (Scaffold: NW_001493188.1) and BTA-86950 (Scaffold: NW_001493187.1). In both maps, these markers map close together, however in our RH map, marker BTA-20131 maps before marker BTA-86950. According to the assembly these markers are approximately 23,000 base pairs away. The log10-likelihood for our order is -3835.05, while the assembly's order log10-likelihood is -3842.27. The second case showed problems in the arrangement within scaffolds. Markers BTA-11589 (NW_001493217.1) and BTA-34555 (NW_001493217.1) show a flip in their positions when compared to the sequence assembly. The log10-likelihood for the assembly, in this case, is -3850.65, while the log10-likelihood for our order is still -3835.03. Both markers are part of the same scaffold indicating a possible mis-assembly within the scaffold.

The inconsistencies observed between our RH map and the assembly cannot be resolved by comparing previously published maps since there are no other maps of BTA14 with a comparable resolution. A complete list of markers showing inconsistent locations when compared to Btau_3.1 is presented in additional file 4.

### Alignment with human chromosome 8

**Figure 3:**
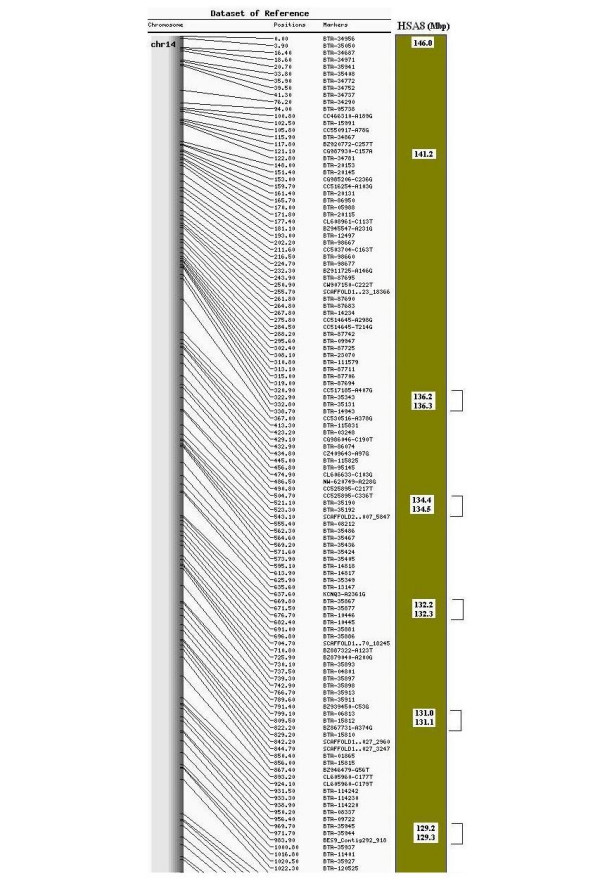
**12 K RH map of BTA14 with Homologous Conserved Synteny Blocks from HSA8**. This figure shows the upper quartile of the map. For the full image please see additional file 6. Comparative map between the 12 K radiation hybrid map of bovine chromosome 14 and human chromosome 8. Human positions are represented in Mbp. Radiation hybrid map distances are scaled in cR. The grey bar represents the chromosome with black lines pointing to the markers. Brackets indicate small inversions, which would otherwise make the order in respect to the human chromosome 8 perfect.

Of the 843 markers ordered on the map, 828 markers (98%) have putative orthologs on the human chromosome 8 (HSA8) (NCBI build 36). Comparative analysis between bovine chromosome 14 and human chromosome 8 identified 4 homologous conserved synteny blocks (HSB): three previously published [4] and an extra conserved synteny block close to the telomere (Figure 3 and additional file 6). This additional HSB block is comprised of 29 markers (BES8_Contig464_1373 to BTA-96554) and lies in a region with high consistency between our RH map and the assembly, therefore confirming the identification of a new evolutionary breakpoint. A number of gaps from a previous published map [4] have been filled and 18 small inversions identified. These inversions were predicted using a set of rules described by Murphy *et al*. [16].

When two or more markers mapped to the same location, their relative positions were decided using a combination of their bovine assembly and human chromosome 8 coordinates, with the bovine coordinates taking precedence over the human ones. In this case, the human coordinates were used to determine whether or not markers appeared in ascending or descending order, depending on which HSB they were in. Once a particular trend was observed, their relative positions were established based on the bovine sequence assembly; meaning that markers were arranged sequentially in an either ascending or descending trend, even if there were disagreements with the human coordinates. For example, according to their human coordinates, the order of the four markers mapping to position 1185.9 cR should be BTA-42142, BTA-42148, BTA-42161 and BTA-42153, however according to their assembly coordinates, BTA-42153 precedes BTA-42161 making the order of all four markers sequential (Additional file 1). Comparative analysis between the bovine sequence assembly and HSA8 resulted in more HSBs than expected (data not shown).

## Discussion

### Comparison with other maps and Btau_3.1

In this study a comprehensive BTA14 RH map was built using the bovine assembly information (Btau_3.1) as a reference order. Traditionally, Lod scores have been used to determine the best fit map; however as the number of markers increases, it becomes more difficult to establish the next best map solely on the basis of these Lod scores. In the comparative method, the best map is a compromise between the RH data and the assembly and it works by comparing the likelihoods and breakpoints for the different maps. Briefly, if two maps have the same likelihood but different breakpoints, the order with fewer breakpoints is preferable. This approach demonstrates extreme robustness when building dense maps, as shown on simulated data and the dog genome [11].

Comparison between our map and the previously released 3 K RH BTA14 map [6] demonstrated a high degree of consistency except for regions where markers were in close proximity. In our map, those markers still map close to each other but with some slight shifts in order, particularly when the positions were just a few centirays apart. Perhaps the resolution of the 3 K panel was not adequate for determining the order for those closely linked markers, since the number of cell lines for this panel is lower (94) than in the 12 K panel (180).

Previously released radiation hybrid maps [4-6] have indicated regions that are inconsistent with the bovine sequence assembly. According to Jann *et al*. [5], BTA14 was not among the chromosomes with a high number of discrepancies with the assembly (Btau_2.0). The inconsistencies observed referred mainly to the assignment of markers to other chromosomes. Such inconsistency still occurred in the latest assembly, but it was most likely due to repeated sequences assigned to multiple chromosomes. McKay *et al*. [6] also indicated incorrectly assigned markers as well as some small inversions in scaffold ordering between their RH map and Btau_2.0 for some chromosomes; confirming that some discrepancies in scaffold arrangement were already present in previous assembly releases. Table 2 summarizes and compares the various BTA14 RH maps.

**Table 2:** Comparison of different BTA14 RH maps including the radiation panel used, techniques used and number of common markers

	McKay *et al *2007	Jann *et al *2006 [5]	This Study
Panel type	WG-3K [2]	WG-3K [2]	WG-12K [8]

Number of markers mapped to BTA14	215	222	843
Number of markers in common with this study	25	0	-
Number of cell lines	94	94	180
Methodologies	Traditional RH approach and Illumina-based RH typing method	Traditional RH approach and conventional RH typing method	Comparative approach and Illumina-based RH typing method

The vast number of markers made available through the bovine sequencing initiative has made possible the compilation of very closely linked markers. However, it is recognized that even this latest assembly contains a possible 20% error in scaffold assembly (George Weinstock, personal communication), with no reports on the specific error rates for the scaffolds discussed here. Mammalian genomes are characterized by large duplications and abundant repetitive sequences which can complicate the final assembly [17]. Finishing a genome does not necessarily indicate that the mis-assemblies will be resolved. It only means that the gaps are closed but that the sequence itself is not confirmed [17]. Software limitations in assembling large, repeated sequences can cause incorrect ordering of large segments of DNA [18].

Differences in the animal resources used to produce the RH map and the bovine assembly for BTA14 seem unlikely to be the cause of the discrepancies discussed here. For instance, a high similarity in marker order should be expected between the genome of the line-bred Hereford bull represented in the BAC map and the genome of his daughter, which was used for the assembly. The pedigree relationship between this sire and daughter is 0.954 (Mike MacNeil, personal communication). However, comparisons between the BAC map, the 12 K BTA14 RH map and the assembly showed that the highest agreement is between the BAC map and the RH map (Warren Snelling, personal communication), with the latter panel being constructed from an Angus bull (JEW38) fibroblast cells [8]. Based on this and the fact that the likelihood for our best map is substantially higher (-3835.03) than the likelihood for the assembly order (-4541.33), the notion that the differences we observed are due to rearrangement of individual animal's genomes seems unlikely.

The ultimate map for a species is the correctly assembled genome sequence with the latest assembly having a 7.1 fold-coverage. The bovine sequence assembly used the whole genome shotgun sequencing approach as well as information from a minimum tiling path of BAC clones across the genome. Contigs, which are referred to as the basic units of contiguous bases, are linked together using information from read pairs at the end of clones. Linked contigs will form scaffolds which are, in turn, arranged along the chromosome using mapping information from MARC 2004 [19] map. Therefore the observed error rate in scaffold arrangement for the assembly is most likely due to the error rate observed in the MARC 2004 linkage map.

A combination of multiple mapping approaches such as linkage and RH maps have demonstrated their feasibility for improving the assembly [20,21]. A number of mapping approaches have aided the arrangement of scaffolds from the first release of the assembly until now [4,19]. Certain high resolution maps such as the one of BTA6 published by Weikard *et al*. [21] presented a gene based comparative radiation hybrid map providing a platform for the assembly. All of these studies have contributed considerable information to the assembly, but mis-assemblies and inconsistencies are still present.

### Comparison with human chromosome 8 (HSA8)

As the density of markers increases, new HSBs and evolutionary breakpoints are likely to be identified through comparative studies. Previously reported HSBs from an independent study [4] are in overall agreement with those reported here. The new HSB identified in our map is supported since marker order in this region is highly consistent with the assembly order [22]. The number of inversions observed in our map (18) was higher than the number identified by a previous BTA14 map (3) [4]. This is not surprising considering the increase in marker density. This increase in marker density coupled with certain limitations of the panel prevented some markers from mapping to unique positions, but by consolidating the human coordinates with the bovine assembly positions for these RH markers with the same position, it was possible to reduce the number of inversions from 25 to 18. A comparative genome assembly approach uses the information from a reference genome to build and arrange the sequenced genome [18]. Therefore, using the high resolution RH map built here in addition to the cattle-human comparative maps already available, it should be possible to resolve rearrangements in the bovine genome assembly.

## Conclusion

With the bovine genome assembly now publicly available, attention has turned from marker discovery towards the proper ordering of these markers. Since the release of early assembly versions, significant improvements have been made through the resolution of mis-assemblies. The integration of different mapping approaches will continue to supply information that can be used to refine the genome sequence. More accurate maps and assemblies will facilitate accurate positioning of QTL affecting economically important traits, haplotype map construction and positional candidate gene searches.

Using high resolution maps, such as the one described in this work, it is possible to identify regions of the bovine genome sequence that can potentially be improved. It is important to acknowledge that all mapping approaches have weaknesses, and that discrepancies between maps are best resolved using information from a variety of sources, such as cattle-human comparative maps or additional sequencing. Through the data presented and discussed here we hope to aid in the generation of subsequent bovine genome assemblies.

## Methods

### Compilation and development of SNP markers on BTA 14

SNPs included in the construction of the RH map were compiled and selected from the Baylor College of Medicine bovine database [15]. At the time the experiment was conducted Btau_2.0 was the latest version available and all markers selected and genotyped were thought to belong to chromosome 14. Additional SNPs were derived from bac end sequences (BES) of the CHORI-240 library [23] and IBISS (Interactive Bovine In Silico SNP) database [24]. The initial selection of markers included 1536 SNP markers, of which 148 were generated either by direct sequencing or by selection from NCBI or IBISS databases. A total of 429 markers could not be genotyped across the RH panel, and therefore were not included in the analysis. The remaining 264 markers were determined to form parts of different linkage groups (different chromosomes) through Lod scores in CarthaGene. A list of SNPs mapped and their respective primer information are given in additional file 1.

### Primer design and sequencing of BES

Primer design for SNPs originating from BES was carried out using primer3 [25] using the following settings (min opt max): primer size: 22 24 26; primer tm: 58 60 62; primer GC%: 40 50 60. Genomic DNA from 12 Angus animals was amplified using a PCR program with initial denaturing for 10 min at 94°C, denaturing for 30 sec at 94°C, annealing (55°C, 60°C or 65°C) and elongation (72°C) for 30 sec in 35 cycles.

PCR products were subjected to a clean up stage consisting of 0.5 ul of an equal mixture of exonuclease I and shrimp alkaline phosphatase enzymes (Invitrogen) for 15 min at 37°C and 15 min at 85°C. Clean PCR products were sequenced using BigDye-terminator chemistry (Applied Biosystems) and a 3730 DNA sequencer (Applied Biosystems). Sequence lengths ranged from 350 bp to 650 bp. The individual SNP sequence data were submitted to GenBank and are publicly available (Table 1). SNPs from Baylor College of Medicine were submitted to Illumina (Illumina, Inc) and passed an internal quality control that predicted complementarity of primers and secondary structures (dimers, hairpin etc.). Only SNPs with an internal score of > 0.6 (out of 1) were selected for genotyping.

### Genotyping

The Illumina BeadStation 5.2 genotyping instrument (Illumina, Inc) was used for high throughput genotyping across the 12 K radiation hybrid panel according to methods described by McKay *et al*. [6]. The software used for the genotyping analysis was Gencall version 5.2 (Illumina, Inc). Loci were scored based on the absence or presence of amplification. Markers that showed amplification in a particular clone were marked as 1, while markers showing no amplification were marked as zero. Markers whose amplification was uncertain were given a score of 2.

### Construction of the 12 K RH BTA14 map

RH analysis was carried out using CarthaGene software package [26]. Previously mapped markers were used to assign markers to cattle chromosome 14 using a LOD score of 14 and a maximum distance of 100. After the linkage analysis was performed 843 markers were determined to be part of one linkage group. Markers were initially analyzed to identify any double markers (same retention pattern). These markers were merged to be part of the same bin. One marker of every bin was then selected to be mapped using the comparative mapping approach. This approach exploits a comparative 2-point model using RH data and the bovine sequence assembly Btau_3.1 as a reference order. This newly developed algorithm incorporated in CarthaGene [10] is described in detail by Faraut *et al*. [11]. The expected number of breakpoints was set to 1 (default setting) and several 2-point reductions (Base TSP+MLE, Extended TSP+MLE, 2-point LOD distance) [27] were solved using the LKH heuristic methods [28]. The final map was further improved by iteratively testing all the marker permutations in a small sliding window of size 7.

### Comparative analysis with of the bovine assembly (Btau_3.1) and human chromosome 8 (HSA8)

Genomic sequence coordinates for SNPs were obtained by performing BLAST [29] comparisons (using an E-value cutoff of 1e-50) between SNP flanking sequences and the latest bovine genome assembly (Btau_3.1). SNPs producing BLAST hits to multiple locations in the bovine genome with the same coverage and sequence identity were removed from CarthaGene's marker input file during RH map construction. Approximate coordinates of the putative orthologous SNP regions in the human genome were obtained by performing BLAST searches (using an E-value cutoff of 1e-3) against the most recent human genome assembly (NCBI build 36). When bovine genome coordinates were available, the 3' end of the 3' flanking sequence of each SNP was extended (using sequence from the bovine genome assembly) prior to performing the comparison with the human genome, to give a total flanking sequence length of 20 kbp. This sequence extension step was performed because the existing flanking sequence did not produce a human genome BLAST hit in most cases. Homologous conserved synteny blocks and inversions between BTA14 and HSA8 were determined according to a set of rules described by Murphy *et al*. [16].

### Graphical representation of BTA14 map and comparative map

Visual representation of map alignments was achieved using AutoGRAPH [30].

## Authors' contributions

EM screened the RH panel, analyzed the RH data, constructed the map and drafted the manuscript. SG constructed the map and resolved issues in the mapping process. PS aligned sequences against the bovine sequence assembly and human chromosome 8 and edited the manuscript. BM performed screening of the RH panel. ZW compiled SNP markers and designed genotyping assay. JW provided the RH panel and SSM initiated and supervised the project and edited the manuscript.

All authors read and approved the final manuscript.

## Supplementary Material

Additional file 1 **Complete list of markers mapped on BTA14**. Summary of markers mapped to 12 K RH BTA14 map, including accession number, RH map position, bovine sequence assembly and human genome coordinates and sequence. Human coordinates that could not be assigned were left blank. Markers highlighted in yellow are those presented in figure 2. Yellow highlighted human coordinates represent small inversions, which if rearranged would be a perfect match with HSA8. BCM-HGSC: Baylor College of Medicine database, IBISS: Interactive Bovine In Silico SNP database, UofA: University of Alberta. Click here for file

Additional file 2 **Retention frequency pattern graph of markers mapped on BTA14**. Graph depicting the range in retention frequency patterns for markers mapped on BTA14 using the 12 K radiation hybrid panel. The dotted blue line represents the average retention frequency value, while the green dotted line represents the line of best fit for the retention frequency values. Click here for file

Additional file 3 **RH genotypes for all markers mapped to BTA14 using the 12 K radiation hybrid panel**. Screening results for the 180 cell lines in the 12 k panel. "0" represents no amplification of the particular marker in the cell line. "1" indicates amplification of the particular marker in the cell line. "2" represents missing results. Click here for file

Additional file 5 Full image of 12 K RH map of BTA14 compared to the corresponding Btau_3.1 map Click here for file

Additional file 4 List of all markers with inconsistent positions when comparing the 12 k RH BTA14 and Btau_3.1 maps Click here for file

Additional file 6 Full image of 12 K RH map of BTA14 with Homologous Conserved Synteny Blocks from HSA8 Click here for file
