# Supplementary figures and images for: A high resolution radiation hybrid map of bovine chromosome 14 identifies scaffold rearrangement in the latest bovine assembly
Source: BMC Genomics. 2007 Jul 26;8:254. doi: 10.1186/1471-2164-8-254 (PMC1959194; doi:10.1186/1471-2164-8-254)

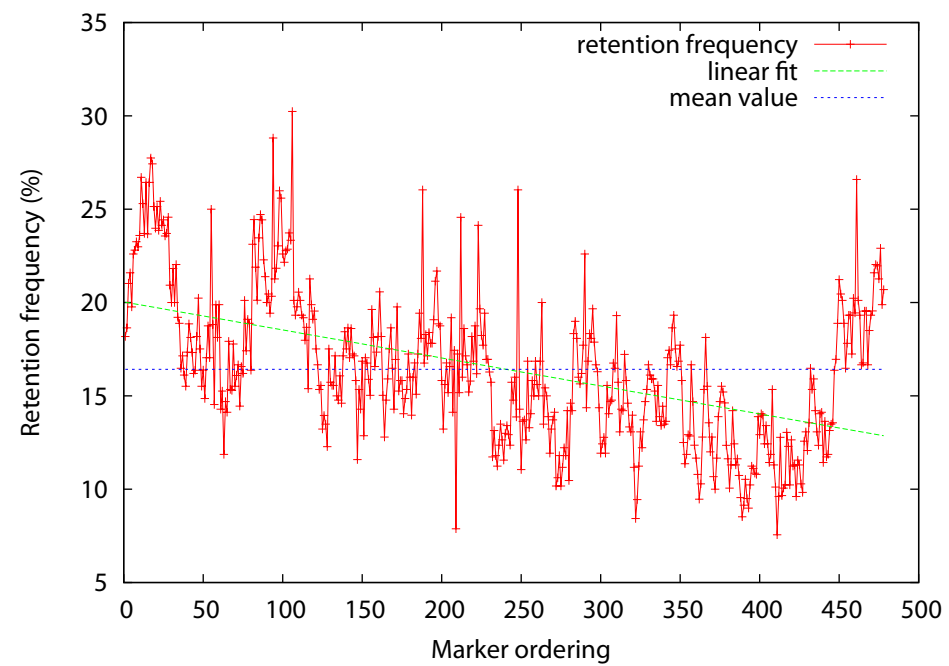

Supplement: Additional file 2 — Retention frequency pattern graph of markers mapped on BTA14. Graph depicting the range in retention frequency patterns for markers mapped on BTA14 using the 12 K radiation hybrid panel. The dotted blue line represents the average retention frequency value, while the green dotted line represents the line of best fit for the retention frequency values. [file 1471-2164-8-254-S2.pdf]

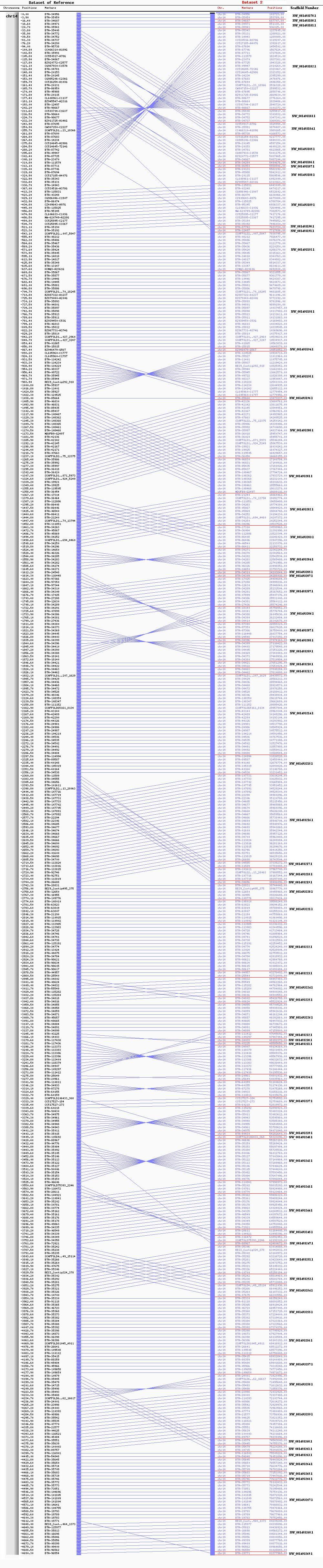

Supplement: Additional file 5 — Full image of 12 K RH map of BTA14 compared to the corresponding Btau_3.1 map [file 1471-2164-8-254-S5.jpeg]

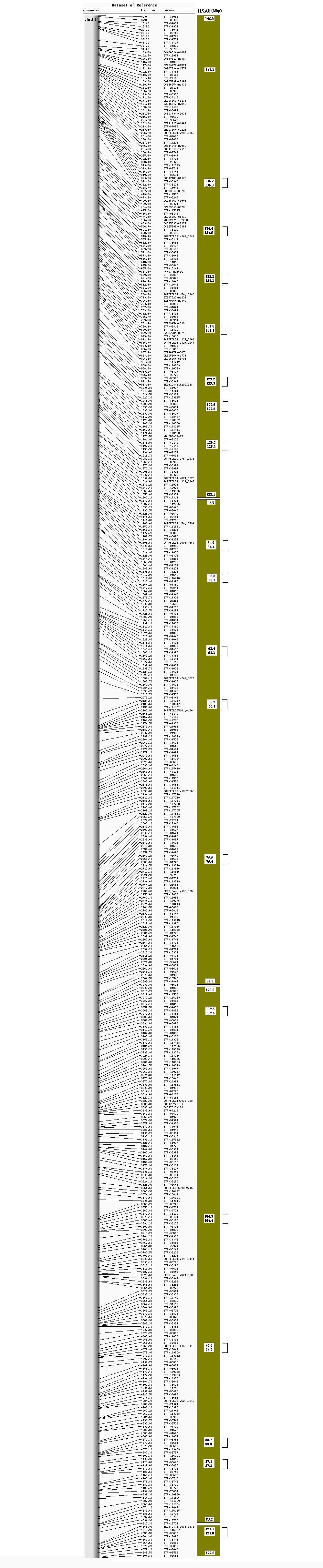

Supplement: Additional file 6 — Full image of 12 K RH map of BTA14 with Homologous Conserved Synteny Blocks from HSA8 [file 1471-2164-8-254-S6.jpeg]
