# Supplementary material for: A high resolution radiation hybrid map of bovine chromosome 14 identifies scaffold rearrangement in the latest bovine assembly
Source: BMC Genomics. 2007 Jul 26;8:254. doi: 10.1186/1471-2164-8-254 (PMC1959194; doi:10.1186/1471-2164-8-254)
Supplement: Additional file 4 — List of all markers with inconsistent positions when comparing the 12 k RH BTA14 and Btau_3.1 maps [file 1471-2164-8-254-S4.doc]

**Markers with inconsistent positions when comparing the 12k RH BTA14 and Btau_3.1 maps.**

| **NCBI Accession number/NCBI SNP id** | **Marker name** | **Discrepancy case (Results Section)** | **BTA14 RH_12,000_ position (cR)** | **Btau_3.1 position (bp)** | **NCBI Accession number (scaffold)** |
| --- | --- | --- | --- | --- | --- |
| ss61480708  ss61480590 | BTA-35408  to  BTA-34737 | A | 33.8  to  41.3 | 3259417  to  3369381 | NW_001493183.1  NW_001493183.1 |
| ss61534608 | BTA-34290 | A | 76.2 | 5490676 | NW_001493186.1 |
| ss61567744  ss69374927 | BTA-95738  to  CC550917-A78G | A,C | 94  to  105.8 | 4166573  to  3824944 | NW_001493184.1  NW_001493184.1 |
| ss61467365  ss69374937 | BTA-34867  to  CC516254-A103G | A,C | 115.9  to  159.7 | 5087204  to  4217777 | NW_001493185.1  NW_001493185.1 |
| ss61476791  ss61563126 | BTA-20131  to  BTA-86950 | A,B | 161.4  to  165.7 | 5582192  to  5558546 | NW_001493188.1  NW_001493187.1 |
| ss38328040  ss69374939 | BTA-05988  to  BZ945547-A231G | A | 170  to  181.1 | 5842011  to  6310161 | NW_001493188.1  NW_001493188.1 |
| ss38334549 | BTA-12497 | A | 193 | 7717936 | NW_001493191.1 |
| ss61497128  ss69374946 | BTA-98667  to  CC514645-T214G | A | 202.2  to  284.5 | 3101373  to  2323067 | NW_001493182.1  NW_001493182.1 |
| ss61494244 | BTA-87742 | A | 288.2 | 7621720 | NW_001493190.1 |
| ss61563555  ss69374951 | BTA-09947  to  CC517185-A407G | A,C | 295.6  to  320.9 | 2010260  to  1392617 | NW_001493182.1  NW_001508604.1 |
| ss61501188  ss69374955 | BTA-35343  To  CC530516-A378G | A | 338.7  to  367 | 1289089  to  1319207 | NW_001493182.1  NW_001493182.1 |
| ss69374978  ss69374979 | CL605960-C177T  to  CL605960-C179T | A | 893.2  to  924.1 | 12779054  to  12779056 | NW_001493195.1  NW_001493195.1 |
| ss61519384  ss61522828 | BTA-114242 to  BTA-120525 | A,C | 931.5  to  1022.3 | 12655112  to  10938710 | NW_001493195.1  NW_001493195.1 |
| ss38328882  btcn20869 | BTA-06830  to  NDUFB9-G249T | A | 1085.9  to  1173.5 | 17140783  to  18689115 | NW_001493201.1  NW_001493201.1 |
| ss61508639  ss61535591 | BTA-42136  to  BTA-36054 | A | 1181.9  to  1358.4 | 13680761  to  16668155 | NW_001493200.1  NW_001493200.1 |
| ss61525638  ss61562835 | BTA-17314  to  BTA-86411 | A | 1367.1  to  1440.6 | 20599967  to  22258733 | NW_001493203.1  NW_001493203.1 |
| ss61477078  ss61570309 | BTA-21240  to  BTA-34296 | A | 1444.8  to  1519.8 | 18869062  to  20507528 | NW_001493202.1  NW_001493202.1 |
| ss38336702  ss61534593 | BTA-14650  to  BTA-34271 | C | 1524  to  1605.6 | 23793721  to  22342243 | NW_001493204.1  NW_001493204.1 |
| ss61570240  ss61496808 | BTA-24548  to  BTA-97344 | A,C | 1612.2  to  1647.1 | 27017291  to  26555321 | NW_001493207.1  NW_001493207.1 |
| ss61534613  ss61478665 | BTA-34310  to  BTA-27436 | A | 1662  to  1799.1 | 24203843  to  25574206 | NW_001493205.1  NW_001493205.1 |
| ss61480485  ss61534647 | BTA-34380  to  BTA-34396 | A,C | 1811.8  to  1840.6 | 27510984  to  27074160 | NW_001493209.1  NW_001493208.1 |
| ss61480499  ss61476316 | BTA-34410  to  BTA-18140 | A,C | 1845.4  to  1872.6 | 26302678  to  25756553 | NW_001493206.1  NW_001493206.1 |
| ss38333641  ss61498127 | BTA-11589  to  BTA-34555 | A,B | 2369.8  to  2383.4 | 37781488  to  37335273 | NW_001493217.1  NW_001493217.1 |
| ss61534788 | BTA-34656 | A | 2385.6 | 38446496 | NW_001493219.1 |
| ss61470582  ss61498127 | BTA-101611  to  BTA-107719 | A | 2393.5  to  2412.8 | 37967708  to  38297010 | NW_001493218.1  NW_001493218.1 |
| ss61515968  ss61530605 | BTA-107731  to  BTA-26688 | A | 2438.5  to  2740.2 | 33614205  to  36743502 | NW_001493216.1  NW_001493216.1 |
| ss61534887  ss38330676 | BTA-34802  to  BTA-08624 | A | 2995.8  to  3001 | 45426787  to  45522923 | NW_001493225.1  NW_001493225.1 |
| ss61480605  ss61480597 | BTA-34832  to  BTA-34816 | A | 3009  to  3042.4 | 44447078  to  44900551 | NW_001493224.1  NW_001493224.1 |
| ss38325277  ss38336085 | BTA-03225  to  BTA-14033 | A,B | 3145  to  3166.1 | 48585654  to  48182373 | NW_001493231.1  NW_001493230.1 |
| ss61521093  ss61534932 | BTA-117438  to  BTA-34907 | C | 3179.6  to  3246.6 | 50195538  to  49134359 | NW_001493232.1  NW_001493232.1 |
| ss61516731  ss61473093 | BTA-109297  to  BTA-111412 | A,B | 3256.8  to  3271.8 | 47947044  to  47645693 | NW_001493229.1  NW_001493229.1 |
| ss69374987  ss61527579 | BTA-25649  to  BTA-20961 | B | 3275.8  to  3277.8 | 50835960  to  50801932 | NW_001493234.1  NW_001493234.1 |
| ss61517555  ss61487100 | BTA-110811  to  BTA-60159 | B | 3301.9  to  3322.7 | 52015677  to  51140625 | NW_001493235.1  NW_001493235.1 |
| rs29013644 | SCAFFOLD106433_368 | A | 3329 | 54323295 | NW_001493240.1 |
| ss61564952  ss61506794 | BTA-90430  to  BTA-115692 | B | 3343.8  to  3409.1 | 54282640  to  52898920 | NW_001507724.1  NW_001493238.1 |
| ss61564183 | BTA-88967 | A | 3428.4 | 62480674 | NW_001493250.1 |
| ss38326828  ss38328658 | BTA-04776  to  BTA-06606 | A,B | 3432.4  to  3525 | 57846844  to  55263181 | NW_001493242.1  NW_001493242.1 |
| rs29022898  ss61520620 | SCAFFOLD75393_2246  to  BTA-116472 | A,B | 3550.6  to  3563.9 | 62192328  to  61892052 | NW_001493247.1  NW_001493247.1 |
| ss61478975  ss61471416 | BTA-28611  to  BTA-104921 | A,C | 3570  to  3582.5 | 61164407  to  61498397 | NW_001493245.1  NW_001493245.1 |
| ss61472941  ss61490518 | BTA-110841  to  BTA-72921 | A | 3601.2 to  3761.8 | 57998973  to  60955585 | NW_001493243.1  NW_001493244.1 |
| ss61535147  ss38329727 | BTA-35242  to  BTA-07675 | A | 3781.1  to  3819.9 | 65514461  to  66203557 | NW_001493253.1  NW_001493254.1 |
| ss61535181  ss38332752 | BTA-35306  to  BTA-10700 | A | 3827.1  to  3943.7 | 63014890  to  65256182 | NW_001493251.1  NW_001493251.1 |
| ss38333031  ss38336767 | BTA-10979  to  BTA-14715 | A | 4190  to  4201.6 | 74543809  to  75391577 | NW_001493260.1  NW_001493261.1 |
| ss61535281  ss61535276 | BTA-35498  to  BTA-24001 | A, C | 4209.8  to  4236.9 | 71858373  to  71420092 | NW_001493258.1  NW_001493258.1 |
| ss61529051  ss61564556 | BTA-23998  to  BTA-89757 | A | 4265.2  to  4382.3 | 72032931  to  74239095 | NW_001493259.1  NW_001493259.1 |
| ss61526929 | BTA-19771 | A | 4612.3 | 81377972 | NW_001493269.1 |

1. Single markers or group of closely mapped markers mapping somewhere else in the bovine sequence assembly.
2. Inversion of flanking markers.
3. Inversion of closely mapped markers
